# Computational decoding of cell-cycle phase effects on cancer hallmarks across breast cancer subtypes

**DOI:** 10.1186/s13058-025-02208-1

**Published:** 2025-12-24

**Authors:** Miguel Castresana-Aguirre, Alexios Matikas, Linda S. Lindström, Nicholas P. Tobin

**Affiliations:** 1 Department of Oncology and Pathology, Karolinska Institutet and University Hospital, BioClinicum, Visionsgatan 4, 171 64 Stockholm, Sweden; 2 Breast Center, Karolinska Comprehensive Cancer Center, Karolinska University Hospital, Stockholm, Sweden

## Abstract

Breast tumors have traditionally been categorized into four subtypes. Recent single-cell advancements, however, reveal that tumors can contain multiple subtypes, reflecting intrinsic heterogeneity. Relatedly, whilst the crucial role of cell cycle regulation in cancer development is widely acknowledged, the impact of cell cycle phases on oncogenic signaling within these subtypes remains unresolved. Here, we examine differences in transcriptomic hallmarks across and within cell cycle phases and breast cancer subtypes in two large single-cell cohorts. We show that accounting for cell cycle phases reveals a broader pool of hallmark pathways while preserving those identified when phases are ignored. Moreover, we pinpoint FDA approved drugs that can target hallmarks within specific phase-subtype combinations. This study highlights the critical role of cell cycle phases in advancing our understanding of cancer biology. The distinct mechanisms observed here suggest that targeting these hallmarks may offer a more precise and effective strategy for patient treatment.

**Supplementary Information:**

The online version contains supplementary material available at 10.1186/s13058-025-02208-1.

## Introduction

Breast cancer accounts for approximately 31% of all female cancers and its incidence has increased by up to 0.5% annually since the early 2000s [1]. Traditional immunohistochemical (IHC) classification of breast cancer is based on the expression of the estrogen receptor (ER), progesterone receptor (PR), and human epidermal growth factor 2 (HER2) receptor. These biomarkers are used to divide breast tumors into luminal (ER+/PR+/−), HER2+ (HER2+, ER+/−, PR+/−) or triple negative (ER−, PR−, HER2−) subtypes. A further sub-division of the luminal subtype can be made with the proliferation marker Ki67 to classify tumors as Luminal A-like (ER+, PR+/−, Ki67 low) and Luminal B-like (ER+, PR+/−, Ki67 high). A genomic profiling approach can also be applied in order to derive breast cancer subtypes that are closely related but not identical to their immunohistochemical counterparts [2, 3]. Applying the commercial version of Prediction Analysis of Microarray 50 (PAM50) signature to gene expression data from a breast tumor results in its classification into one of four main subtypes: Luminal A, Luminal B, Her2-enriched, Basal-like [4]. However, due to the intrinsic heterogeneity of breast tumors, classifying a whole tumor uniformly may not capture their biological complexity [5–8], a task that is better suited to single-cell RNA-sequencing (scRNA-seq) analyses [9]. scRNA-seq has enabled the analysis of the transcriptome at a single-cell level, providing a detailed view of the cellular landscape within tumor cell populations [10, 11]. Recently, the bulk tumor PAM50 approach has been further developed in order to enable its application as a single-cell classifier (scPAM50) that can assign individual cancer epithelial cells to one of four breast cancer subtypes: Luminal A, Luminal B, Her2, and Basal [5]. In addition to this heterogeneity in single-cell molecular subtypes, a second seldom considered layer of complexity is that of tumor cell cycle phase.

Dysregulation of the cell cycle is a hallmark of cancer [12], uncontrolled cell proliferation, and tumor growth [13]. There are also clear differences in cell cycle activity levels between normal and tumor tissues [14], making the cell cycle a key target for cancer therapy [15–17]. Previous studies have shown that cells in different cell cycle phases show different transcriptomic profiles [18–21]. This is in line with the key concept that biological signaling pathways can be modulated by a cell’s progress through the cell cycle [22, 23]. Relatedly, several studies have shown that understanding the interplay between cell cycle regulation and oncogenic signaling pathways is crucial for developing targeted therapies in breast cancer both at a bulk [24, 25] and single-cell level [26–29]. To date however, studies examining the differences in pathways or cancer hallmarks as a cell proceeds through the cell cycle are lacking. To this end, we present a comprehensive single-cell analysis, in two large public cohorts, that aims to understand the critical role of the cell cycle in breast cancer. We determine expression differences in cancer hallmarks across the G0/G1, S and G2/M cell cycle phases and molecular subtypes before placing our results in the context of the potential implications for targeted therapy.

## Material and methods

### Data processing and single-cell annotations

Two previously published single-cell breast cancer atlases were used for this study: cohort-1 containing 100,064 cells from 26 patients [5] (GSE176078) and cohort-2 containing 208,788 cells from 32 patients [30] (GSE161529). Cohort-2 was used to replicate the analysis performed in cohort-1, ensuring the robustness and reproducibility of our findings across independent datasets. We excluded normal tissue, male samples, and involved-lymph-node samples according to the source metadata. Whole genome scRNA-seq data with 20,224 annotated genes and basic clinico-pathological information was available for both cohorts. Cells were filtered out if fewer than 200 genes were detected in a cell or if their mitochondrial content was higher than 20%. The cohorts were log-normalized with a scaling factor of 10,000. Cell cycle phases (G0/G1, S, G2/M) were inferred using the Seurat package in R (RRID:SCR_001905) version 4.4.2 with predefined cell cycle markers [31]. In this study, we focused solely on cancer epithelial cells. This classification had already been made by the original authors in cohort-1 and totaled 24,489 cancer epithelial cells. For cohort-2, we inferred the cell types using the SingleR tool [32], having first trained and tested the model on cohort-1. This approach classified 95,401 cells as cancer epithelial in cohort-2. scPAM50 subtypes were inferred for cancer epithelial cells using the scSubtype tool [5] (https://github.com/Swarbricklab-code/BrCa_cell_atlas/tree/main/scSubtype) to classify individual cells as Luminal A (LumA), Luminal B (LumB), HER2-enriched (Her2), and Basal-like (Basal). Of note, Luminal B tumors may be HER2-positive by IHC, reflecting receptor overexpression/amplification, whereas the PAM50 HER2-enriched subtype is a distinct transcriptional class that typically lacks strong ER-driven programs. We use this genomic (PAM50/scPAM50) definition throughout when referring to ‘HER2-enriched’.

### Differential Gene Expression (DGE) analysis and comparisons

As differential expression comparisons span cells across all tumors within cohort-1 and cohort-2 (separately), addressing the confounding effect of tumor variation seems crucial. A recent comprehensive benchmark demonstrated, however, that for cohorts characterized by low depth (~ 4%), high sparsity (~ 90%), and significant batch effects, data integration could be detrimental and is not recommended [33]. Therefore, we selected the best performing approach from this benchmark which was a covariate model for breast cancer subtypes and cell cycle phases using LimmaTrend, LIMMA (RRID:SCR_010943) R package v. 3.52.2 [34], which is more suited to our cohort’s characteristics. We applied a two-tailed, unpaired moderated t-test to identify genes exhibiting significant upregulation or downregulation, and genes were ranked based on their resulting t-statistics.

**Fig. 1:**
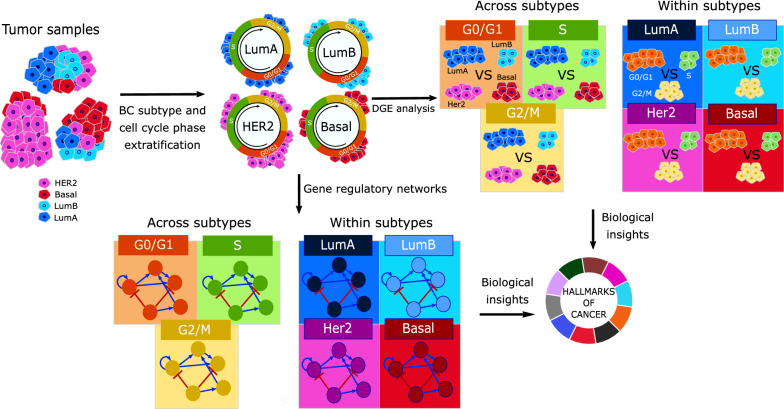
Overview of the study. Starting from patient tumor samples, we infer breast cancer subtype and cell cycle phase per cell. Then, in the *across subtype* comparison, we perform differential expression analysis of breast cancer subtypes within cell cycle phases and infer a gene regulatory network per cell cycle phase. Finally, we look for biological insights by analyzing the hallmarks of cancer gene sets. In the *within subtype* comparison, we perform differential expression analysis between cells of different cell cycle phases within breast cancer subtypes, and we infer a gene regulatory network per subtype

In line with our aim to map the differences in cancer hallmarks across cell cycle phases taking breast cancer subtype into account, two distinct analysis approaches were adopted. In the first approach, we derived DGE lists by comparing cells from LumA, LumB, Her2 or Basal subtypes against each other within the same cell cycle phase (e.g., LumA G0/G1 cells vs. LumB G0/G1 + Her2 G0/G1 + Basal G0/G1 cells). In the second approach, we derived DGE lists by comparing cells from G0/G1, S or G2/M cell cycle phases against each other within the same scPAM50 subtype (e.g., LumA G0/G1 cells vs. LumA S + LumA G2/M). For clarity, we termed these analyses “*across subtype*” and “*within subtype*”, respectively, and they are presented diagrammatically in Fig. 1. Since this study analyzed publicly available single-cell transcriptomic data, group assignments were based on predefined biological annotations rather than randomization. Cells were classified into groups according to their inferred cell cycle phase and breast cancer subtype.

### Gene regulatory networks (GRNs)

GRNs were generated using pySCENIC (RRID:SCR_025802), a Python implementation of SCENIC (RRID:SCR_017247) [35], which resolves gene-to-gene interactions and gene co-expression modules. SCENIC identifies regulons that are composed of a transcription factor (TF) and its directly regulated genes by searching for over-represented DNA motifs near target genes. Thus, suggesting regulatory interactions and emphasizing biologically plausible relationships. The whole genome was used for GRN inference and the regulon activity in each cell was calculated using AUCell (RRID:SCR_021327) implemented on SCENIC. For within subtype analysis, four GRNs were inferred—one for each breast cancer subtype, and for across subtype analysis, three GRNs were delineated—one for each cell cycle phase. For example, the Luminal A GRN includes all cells of that subtype across all cell cycle phases. For each analysis, we computed AUCell per cell and summarized group-level regulon activity by the mean AUCell across cells in that group. Regulon–group pairs were retained if their cluster-averaged AUCell was at the global 95th percentile across all regulon–group pairs (Gopalan et al. 2021). Regulon specificity was quantified using the Regulon Specificity Score (RSS), with a threshold of RSS > 0.20 (Hiltensperger et al., 2021); the full RSS distributions are shown in Supplementary Fig. 1.

### Pathway enrichment analysis

We focused on the hallmarks of cancer pathways from the Molecular Signatures Database (MSigDB) (RRID:SCR_016863) as the pivotal biological mechanisms underlying cancer development and progression. To gain insights into the relationship between the DGE lists and the MSigDB cancer hallmarks, we used Fast Gene Set Enrichment Analysis (FGSEA) (RRID:SCR_020938) [36], reporting NES, *p*-value, and FDR. Pathways were considered enriched if NES > 0 and FDR < 0.05 and depleted if NES < 0 and FDR < 0.05. For the regulons inferred from the GRNs, the over-representation analysis (ORA) in the R package clusterProfiler (RRID:SCR_016884) version 4.8.3 [37] was utilized, analyzing if regulons significantly overlap with pathways of interest. To account for multiple testing, we applied the Benjamini–Hochberg procedure for correcting *p* values [38]. Throughout the paper, when we mention enriched or depleted pathways, they are statistically significant (FDR < 0.05).

### Transcription factor targets and candidate compounds

To identify potential therapeutic targets and candidate compounds, we integrated two curated resources: The Comparative Toxicogenomics Database (CTD)(RRID:SCR_006530) [39], focusing on curated chemical targets relevant to *Homo sapiens* and specific to breast cancer, with an emphasis on mRNA-based compounds, and DrugBank (RRID:SCR_002700) [40] FDA approved drugs for *Homo sapiens*. The number of candidate compounds is 2106 and 1607 for CTD and DrugBank, respectively. Compound targets were selected according to the following criteria: TFs from the regulons identified in the GRNs that have associated compound candidates, regulons with an RSS > 0.2, and regulons with AUCell above the 95th percentile.

### Statistical analysis

All analyses were performed using R (RRID:SCR_001905) version 4.4.2 and Python (RRID:SCR_008394) version 3.9.19. Given that this study is based on publicly available scRNA-seq datasets, no formal power analysis was performed to determine sample size a priori. Instead, all available cells within cohort-1 and cohort-2 were included to maximize statistical power. To compare the proportion of cell cycle phases to scPAM50 subtypes we used a Chi-square test for independence and a Cramer V test using the stats and vcd R packages, respectively. This analysis assumes that both variables are categorical and mutually exclusive, that observations are independent, and that all expected cell frequencies meet the assumption of being ≥ 5. To ensure robustness for pathway enrichment and compound targets-candidate analyses, results were only considered significant if the pathway/ compound was identified in the same phase and subtype in both cohorts. *P*-values from all hypothesis-testing procedures were adjusted for multiple testing using the Benjamini–Hochberg method, with FDR < 0.05 considered statistically significant.

Code to reproduce the results of this study is publicly available at https://github.com/MiguelCastresana/bc_decoding_cellcycle

## Results

### scPAM50 and cell cycle phase proportions

**Fig. 2:**
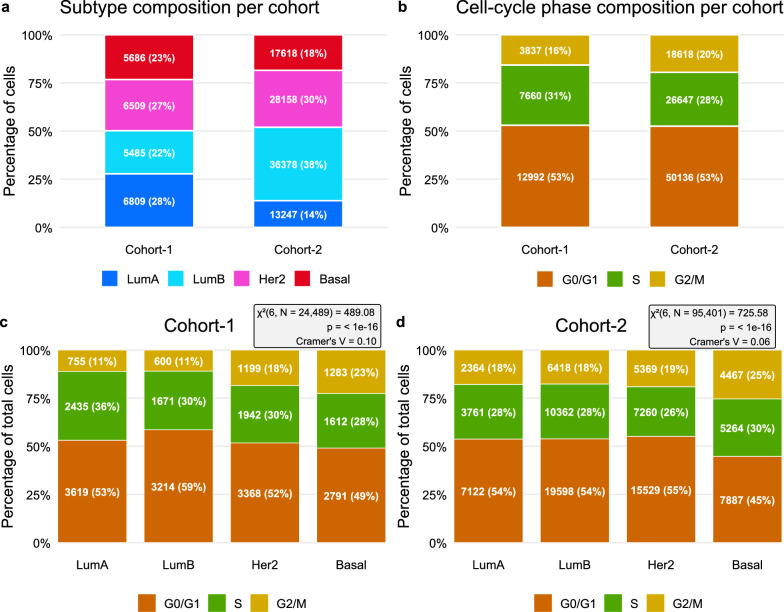
Subtype and cell-cycle phase composition of single cancer epithelial cells in cohort-1 and cohort-2. **a** Stacked bar charts showing the proportion of scPAM50 subtypes (LumA, LumB, Her2, Basal; corresponding to Luminal A, Luminal B, HER2-enriched, and Basal-like) within each cohort. **b** Stacked bar charts showing the proportion of cells in each cell-cycle phase (G0/G1, S, G2/M) within each cohort. **c**–**d** Within each cohort, cells were pooled according to scPAM50 subtype and then split by cell-cycle phase, yielding phase composition for each subtype. Numbers inside segments indicate the number of cells and the within-bar percentage. Boxes report the χ^2^ test statistic, *p*-value, and Cramér’s V as an effect-size measure for the association between subtype and cell-cycle phase. Only cells passing QC were included

The scSubtype classifier was used to classify the single cancer epithelial cells of cohort-1 (N = 24,489) and cohort-2 (N = 95,401) into four breast cancer subtypes. In cohort-1, 6,809 (28%) individual cells were classified as LumA, 5,485 (22%) as LumB, 6,509 (27%) as Her2 and 5,686 (23%) as Basal (Fig. 2a, left bar). In cohort-2, 13,247 (14%) cells were LumA, 36,378 (38%) LumB, 28,158 (30%) Her2, and 17,618 (18%) Basal (Fig. 2a, right bar). We first compared the proportions of scPAM50 subtype calls within individual tumors to their corresponding IHC classifications. Generally, the highest proportion of single-cell subtype calls within each tumor aligns with its IHC across both cohort-1 and cohort-2 (Supplementary Fig. 2a and b, respectively). For example, the first tumor CID3941 (Supplementary Fig. 2a) is classified as ER+ by IHC, and the most frequent/highest proportion of single cells are classified as LumA (53%) by the scSubtype predictor. Luminal A tumors have been previously shown to be predominantly ER+ [3], In cohort-2, LumB was more frequently identified as the highest proportion of single-cell subtypes within individual tumors and we observe an overall matching between IHC and the most frequent sc-subtypes for ER+, HER2 +, and TNBC tumors.

In addition to subtypes, single cells were also categorized into three main cell cycle phases G0/G1, S, and G2/M. Overall, in cohort-1, 12,992 (53%) were found to be in the G0/G1 phase, 7,660 (31%) in S, and 3,837 (16%) in G2/M (Fig. 2b, left bar). In cohort-2, 50,136 (53%) of cells were in G0/G1, 26,647 (28%) in S, and 18,618 (20%) in G2/M (Fig. 2b, right bar). We also assessed single cell—cell cycle phases in relation to scPAM50 subtypes and found a statistically significant association in both cohort-1 (χ2(6, N = 24,489) = 489.08, *p* < 0.0001, Fig. 2c) and cohort-2 (χ2(6, N = 95,401) = 725.58, *p* < 0.0001, Fig. 2d). However, Cramér’s V tests with values of 0.10 and 0.06, respectively, suggest that the association is weak. This implies that while the relationship is statistically significant, it only accounts for a small proportion of the variability between the cell cycle phases and scPAM50 subtypes.

### Differential gene expression analysis identifies altered subtype and cell cycle phase specific cancer hallmark pathways

#### Gene set enrichment: across subtype cancer hallmark pathway analysis

**Fig. 3:**
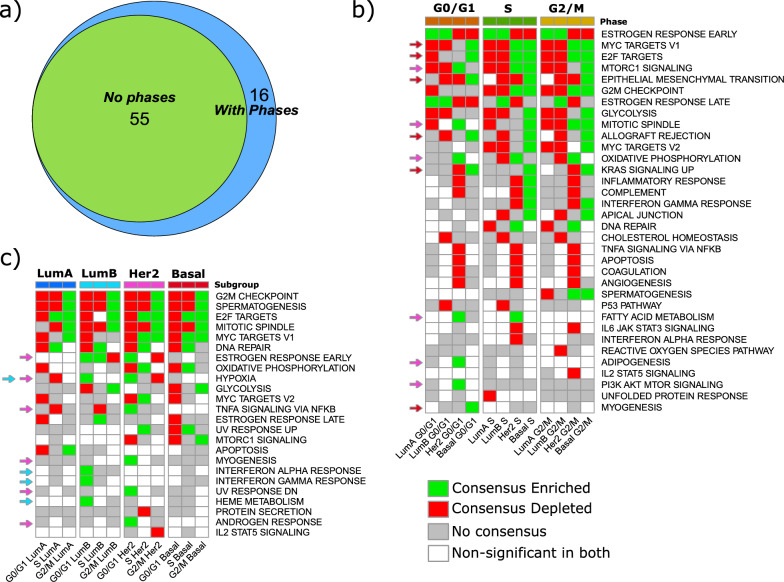
Pathway enrichment analysis by subtype and cell-cycle phase using FGSEA **a** Venn diagram showing counts of significant subtype–pathway associations identified by FGSEA at FDR < 0.05 when subtypes are analyzed without phase and when analyzed with phase stratification. With phases, each subtype–pathway pair is tested in G0/G1, S, and G2/M; it contributes one association if significant in any phase. **b** *Across subtype* comparison, cells in the same cell cycle phase but in different breast cancer subtypes are compared (e.g., Luminal A against Luminal B, Her2, and Basal in G0/G1 phase). **c** *Within subtypes* comparison, cells in the same breast cancer subtype are compared (e.g., Luminal A in G0/G1 against Luminal A in S and Luminal A in G2/M). Heatmap encoding (**b**–**c**): each tile summarizes two-cohort consensus: green = Consensus Enriched (NES > 0 and FDR < 0.05 in both cohorts), red = Consensus Depleted (NES < 0 and FDR < 0.05 in both cohorts), gray = No consensus (not significant in at least one cohort), white = Non-significant in both. Only pathways with at least one consensus call are shown. Colored arrows mark pathways uniquely enriched in G0/G1 within a single subtype. Arrow colors by subtype: dark blue = Luminal A, light blue = Luminal B, pink = Her2, red = Basal (e.g., the red arrow highlights a pathway enriched only in Basal G0/G1, not in LumA/LumB/Her2 G0/G1)

Next, we aimed to determine if taking cell cycle phases into account resulted in the identification of additional differentially expressed cancer hallmarks relative to not taking phases into account. For this, we conducted bulk comparisons *across subtypes* without accounting for cell cycle phase (e.g., all single LumA cells from all tumors versus all single LumB, Her2, and Basal cells). The same analysis was then repeated, comparing each subtype to the remaining three, but this time the same cells were separated into both single-cell subtypes and G0/G1, S, and G2/M cell cycle phases (e.g., LumA G0/G1 vs. LumB G0/G1 + Her2 G0/G1 + Basal G0/G1). In total, bulk analysis identified 55 significant subtype–pathway associations at FDR < 0.05. Repeating the analysis with phase stratification yielded 71 significant subtype–pathway associations that were significant in at least one phase. As depicted in the Venn diagram in Fig. 3a, all subtype–pathway associations identified in bulk analysis are also present in phase-specific instances. This indicates that unique biological information emerges from subtype comparisons that incorporate cell cycle phase distinctions.

Focusing specifically on these subtype-pathway pairs, when stratified by cell cycle phase (see full list in Supplementary Table 1), we observed biological signaling commonalities regardless of the individual cell cycle phase being analyzed. Specifically, Her2 and Basal cells had more enriched pathways relative to LumA/B cells in all phases (Fig. 3b, compare subtype columns in G0/G1, S, and G2/M phases, green = enriched, red = depleted). The ESTROGEN_RESPONSE_EARLY pathway was enriched in LumA/B and depleted in Her2 and Basal subtype cells in all phases. MYC_TARGETS_V1, E2F_TARGETS and G2M_CHECKPOINT hallmarks were depleted in LumA/B and enriched in Her2 and Basal subtype cells in S and G2M phases. Her2 cells exhibit a significant enrichment of OXIDATIVE_PHOSPHORYLATION, and depletion of INFLAMMATORY_RESPONSE, COMPLEMENT, TNFA_SIGNALING_VIA_NFKB, APOPTOSIS, COAGULATION, and ANGIOGENESIS pathways in all cell cycle phases, whilst Basal cells show the highest enrichment of pathways related to EPITHELIAL_MESENCHYMAL_TRANSITION, ALLOGRAFT_REJECTION, and KRAS_SIGNALING_UP in all phases.

As G0/G1 cells likely contain a subpopulation of cells that may be treatment resistant [41, 42], it is pertinent to highlight enriched hallmarks that could form the basis of a targeted treatment in this phase. Aside from the enrichment of estrogen response pathways noted above for LumA and B cells, we also found that Her2 cells show enrichment for MTORC1_SIGNALING, MITOTIC_SPINDLE, OXIDATIVE_PHOSPHORYLATION, FATTY_ACID_METABOLISM, ADIPOGENESIS, and PI3K_AKT_MTOR_SIGNALING hallmarks (Fig. 3b, see pink arrows on the left-hand side). Basal cells showed enrichment of MYC_TARGETS_V1, E2F_TARGETS, EPITHELIAL_MESENCHYMAL_TRANSITION, ALLOGRAFT_REJECTION, KRAS_SIGNALING_UP and MYOGENESIS (Fig. 3b, red arrows).

#### Gene set enrichment: within subtype cancer hallmark pathway analysis

In our initial analyses, we studied DGE focused on the differences *across subtypes* when considering the same cell cycle phase. Next, we studied DGE by comparing each cell cycle phase to the remaining two *within* the same subtype (see full list in Supplementary Table 1).

This analysis highlighted biological signaling commonalities regardless of the individual subtypes being analyzed. G2M_CHECKPOINT, SPERMATOGENESIS and MITOTIC_SPINDLE were all depleted in G0/G1 and S-phases and enriched in G2/M cells for all four subtypes, except for MITOTIC_SPINDLE for LumA G0/G1. Similarly, the MYC_TARGETS_V1 hallmark was depleted in G0/G1 and enriched in G2/M cells for LumA, Her2, and Basal subtypes but interestingly, it was not enriched for LumB G2/M (Fig. 3c). In addition, we found that whilst a pathway can show enrichment *within* the same subtype when comparing its cell cycle phases to each other, it can still be depleted when comparing *across* subtypes. The E2F_TARGETS pathway provides a good example of this. When examining changes between cell cycle phases *within* LumA cells, we found this pathway to be depleted in the G0/G1 phase relative to the S and G2/M phases but enriched in the S (vs. G0/G1 + G2/M) and G2/M (vs. G0/G1 and S) phases (Fig. 3c, see “E2F_TARGETS” in the heatmap). When comparing *across* subtypes, however, we found the pathway to be depleted in LumA cells in all cell cycle phases relative to LumB, Her2, and Basal cells (Fig. 3b, see “E2F_TARGETS”). Put simply, while a pathway can be depleted in one subtype when compared *across* all other subtypes, it can still show differences in depletion and enrichment when comparing cell cycle phases *within* the same subtype.

Finally, we again highlight hallmarks that are enriched in the G0/G1 phase. We found that HYPOXIA is enriched in this phase in LumB and Her2 cells. In LumB cells alone we found INTERFERON_ALPHA, INTERFERON_GAMMA, and HEME_METABOLISM enriched. Finally, in Her2 cells alone we found ESTROGEN_RESPONSE_EARLY, TNFA_SIGNALING_VIA_NFKB, MYOGENESIS, UV_RESPONSE_DN, and ANDROGEN_RESPONSE enriched. (Fig. 3c, see colored arrows on the left-hand side in combination with the subtype group at the top of the plot. Light blue arrows = LumB cells, pink = Her2 cells).

### GRN analysis

Differential gene expression analysis identifies genes with statistically significant expression variations across different conditions or groups. GRNs are a complementary methodology that aims to disentangle the complex interplay of these genes within networks. Thus, while DGE pinpoints genes of interest, GRNs provide the contextual and mechanistic insights necessary for developing targeted therapies.

Using the multi-step filtering process (see Methods), we obtained a compact set of regulons that were both active and pathway-supported in each cohort (Supplementary Fig. 3). Across subtypes, this yielded 45 pathway-supported regulons in cohort-1 and 49 regulons in cohort-2, with an intersection of 11 regulons. Within subtypes, we retained 41 pathway-supported regulons in cohort-1 and 40 regulons in cohort-2, of which 19 regulons were shared between cohorts.

#### GRN: across subtype cancer hallmark pathway analysis

**Fig. 4:**
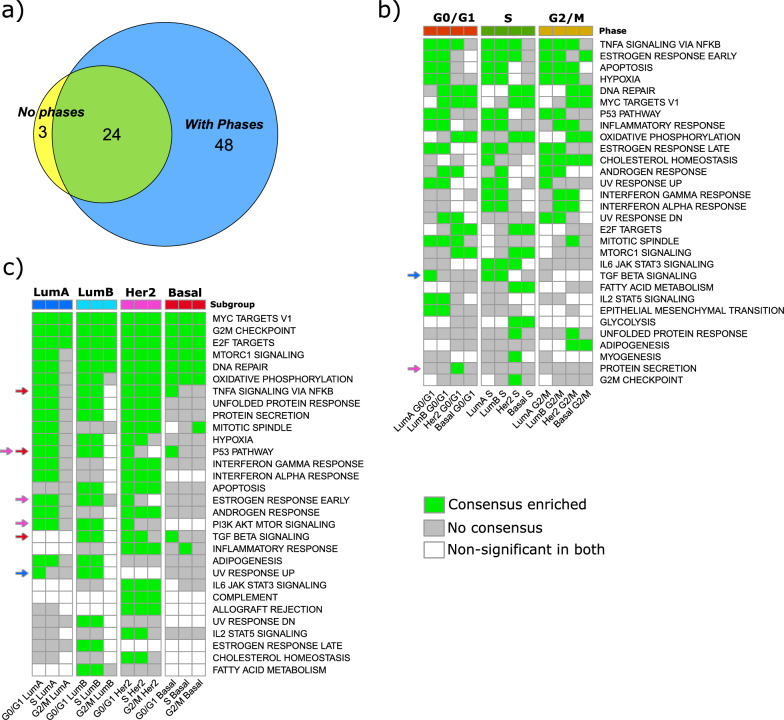
Pathway enrichment analysis of the regulons derived from the GRNs by subtype and cell-cycle phase using over-representation analysis (ORA). **a** Venn diagram showing counts of significant subtype–pathway associations at FDR < 0.05 when subtypes are analyzed without phase and with phase stratification. With phases, each subtype–pathway pair is tested in G0/G1, S, and G2/M; it contributes one association if significant in any phase. **b** Across subtype comparison, a GRN is inferred for cells in the same cell cycle phase but in different breast cancer subtypes (e.g., all cells in G0/G1 phase for all breast cancer subtypes). **c** Within subtype comparison, a GRN is inferred for cells in the same breast cancer subtype but in different cell cycle phases (e.g., all cells in Luminal A for all cell cycle phases). Heatmap encoding (**b**–**c**): each tile summarizes two-cohort consensus: green = Consensus Enriched (FDR < 0.05 in both cohorts), gray = No consensus (not significant in at least one cohort), white = Non-significant in both. Only pathways with at least one consensus call are shown. Colored arrows mark pathways uniquely enriched in G0/G1 within a single subtype. Arrow colors by subtype: dark blue = Luminal A, light blue = Luminal B, pink = Her2, red = Basal (e.g., the red arrow highlights a pathway enriched only in Basal G0/G1, not in LumA/LumB/Her2 G0/G1)

Similarly to our FGSEA analysis, we initially conducted bulk comparisons *across subtypes* without accounting for cell cycle phase. In total, bulk analysis identified 27 significantly differentially expressed cancer hallmark pathways when stratified by subtype alone, while phase-specific stratification identified 72. Of these, 24 subtype-pathway hits overlapped with bulk analysis, whereas 3 and 48 subtype-pathway combinations were unique to bulk and across subtype analyses, respectively (Fig. 4a). This indicates again, as DGE analysis did, that unique biological information emerges from subtype comparisons that incorporate cell cycle phase distinctions.

Next, we again examined the biological signaling commonalities found across all cell cycle phases (Fig. 4b). Note that in our GRN analysis, we used ORA to determine pathways that statistically overlap with the regulons of interest. TNFA_SIGNALING_VIA_NFKB, ESTROGEN_RESPONSE_EARLY, APOPTOSIS, HYPOXIA, P53_PATHWAY, and ESTROGEN_RESPONSE_LATE hallmarks were statistically significant in both LumA and LumB cells in all cell cycle phases. For Her2 and Basal cells, DNA_REPAIR, MYC_TARGETS_V1, and OXIDATIVE_PHOSPHORYLATION hallmarks were statistically significant in all phases.

Focusing again on the G0/G1 phase, examples of pathways unique to a subtype in G0/G1 included TGF_BETA_SIGNALING in LumA cells and PROTEIN_SECRETION in Her2 cells.

#### GRN: within subtype cancer hallmark pathway analysis

The *within* subtype GRN analysis showed, for example, that the MYC_TARGETS_V1, G2M_CHECKPOINT, and E2F_TARGETS pathways were significant in all subtypes for all cell cycle phases. MTORC1_SIGNALING and DNA_REPAIR were significant in all phases in LumB, Her2, and Basal cells but not in LumA G2/M (Fig. 4c).

As previously, we again highlight hallmarks that are enriched in the G0/G1 phase. We found P53_PATHWAY to be statistically significant for Her2 and Basal cells in this phase. In LumA cells, we found UV_RESPONSE_UP. In Her2 cells alone, we found ESTROGEN_RESPONSE_EARLY and PI3K_AKT_MTOR_SIGNALING. Finally, in Basal cells alone, we found TNFA_SIGNALING_VIA_NFKB and TGF_BETA_SIGNALING (Fig. 4c, see colored arrows on the left-hand side in combination with subtype groups at the top of the plot).

### Transcription factor targets and candidate compounds

**Fig. 5:**
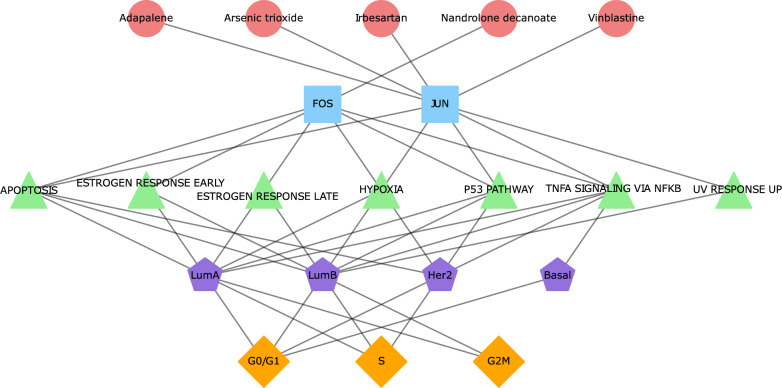
A multipartite network with five distinct levels: FDA approved drugs from DrugBank, transcription factor (regulon), hallmark pathway, breast cancer subtypes, and cell cycle phases. Associations are represented as links

**Fig. 6:**
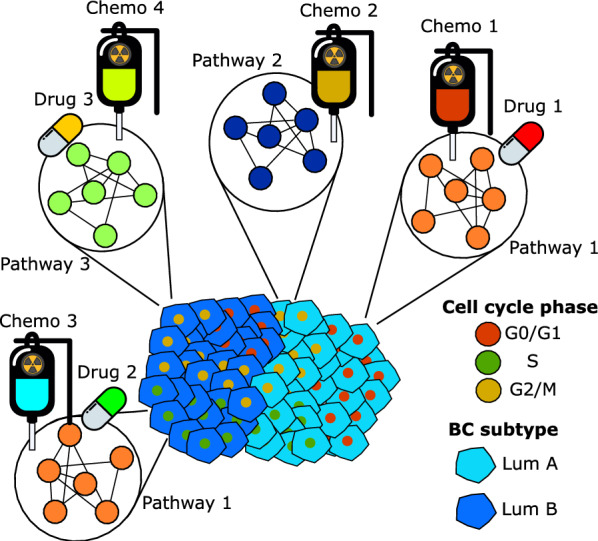
Example of potential applications of subtype- and cell cycle phase-specific therapies for a heterogeneous breast cancer tumor. Distinct tumor regions could be targeted with personalized combinations of drugs and chemotherapeutic agents, each selected based on the tumor’s breast cancer subtypes and specific cell cycle phases

Finally, we sought to identify potential compounds from both *across* and *within* subtype GRN analyses for specific subtype-phase enriched pathways. Our analysis found 5 drugs and 7 chemicals from the DrugBank and CTD databases, respectively, that target the same TF (regulon)-subtype-phase-pathway combination in both cohorts. As multiple drugs/chemicals can target the same combination we provide examples here of FDA approved drugs only for each hallmark. The complete list of compounds-TF(regulon)-pathway-subtype-phase combinations is presented in Supplementary Table 2, and in the form of multipartite network plots in Fig. 5 (for DrugBank) and Supplementary Fig. 4 (for CTD). The DrugBank drugs we identified targeted two main TFs (FOS and JUN) in LumA, LumB and Her2 cells, whereas in Basal cells only JUN was identified. Specifically, in the G0/G1 phase, drugs were found for the TNFA_SIGNALING_VIA_NFKB hallmark (e.g., Adapalene or Vinblastine) in all four breast cancer subtypes, for the ESTROGEN_RESPONSE_EARLY and ESTROGEN_RESPONSE_LATE hallmarks (e.g., Nandrolone decanoate) in LumA and LumB cells, for the P53_PATHWAY (e.g., Irbesartan) in LumB and Her2 cells and for UV_RESPONSE_UP (e.g., Adapalene) in LumB cells only. In the S phase, 4 targetable pathways were found for both LumA and B cells: APOPTOSIS, P53_PATHWAY, TNFA_SIGNALING_VIA_NFKB, and HYPOXIA (e.g., all targetable with the drug Irbesartan). Additionally, UV_RESPONSE_UP (e.g., Vinblastine) can be targeted in S phase LumB cells, as can the TNFA_SIGNALING_VIA_NFKB pathway (e.g., Irbesartan) in Her2 cells. Finally, for the G2/M phase, TNFA_SIGNALING_VIA_NFKB and APOPTOSIS (e.g., Nandrolone decanoate) were found to have significant drugs for both LumA and LumB cells. The CTD chemicals we identified targeted three main TFs (FOS, JUN and FOXM1) in all subtype cells and all chemicals along with the specific TF(regulon)-pathway-subtype-phase combinations they target are shown in Supplementary Table 2 and Supplementary Fig. 4. Taken together, these results demonstrate the possibility of identifying and targeting key hallmarks within specific subtype-phase combinations. This may enhance future precision oncology initiatives and promote the use of combinational or sequential therapies for patient treatment (an illustrative example is shown in Fig. 6 and discussed further below).

## Discussion

In this study, we examined the differences in cancer hallmark signaling pathways across cell cycle phases taking breast cancer subtypes into account. Our analysis involved two distinct approaches: comparing cells *across* different breast cancer subtypes that are in the same cell cycle phase and comparing cells *within* the same subtype that are in different cell cycle phases. In general, we found that taking cell cycle phases into account identifies a larger pool of relevant hallmark pathways that are not found when cell cycle phases are ignored. We showed that pathways that are enriched/depleted in one subtype when comparing *across* subtypes can also be enriched/depleted in one cell cycle phase relative to others when comparing *within* a single subtype (e.g., E2F_TARGETS in Luminal A). We also identified pathways that were uniquely enriched in specific subtype and cell cycle phases (e.g., MTORC1_SIGNALING and KRAS_SIGNALING_UP in G0/G1 HER2 and Basal cells, respectively). Finally, we provide examples of chemicals and FDA approved drugs that can target transcription factors upstream of relevant pathways e.g., the drug Vinblastine targets the transcription factor JUN, which regulates a regulon whose genes significantly overlap with those in APOPTOSIS, P53_PATHWAY, TNFA_SIGNALING_VIA_NFKB, and HYPOXIA in G0/G1 phase Her2 cells.

Genomic analyses of the signaling pathways and cellular processes active in tumors have traditionally been performed on bulk samples. This means that the expression of genes across a broad range of heterogeneous cells is averaged and the signal coming from pathways that are differentially expressed in subgroups of cells can be diminished or lost. Recently, a better understanding of this heterogeneity has been facilitated through single-cell [5, 30, 43–45] and spatial [5, 46–49] transcriptomic analyses. Building on this work, our analyses found both homogeneity and heterogeneity in the cancer hallmarks expressed between breast cancer subtypes and between cell cycle phases. Placing these findings in the context of published literature is challenging owing to previous studies having only compared hallmark pathways between bulk tumor molecular subtypes, however, some notable parallels can be drawn. In general, we found that LumA and LumB cells showed similarity in depleted pathways relative to Her2 and/or Basal cells. Both the MYC_TARGETS_V1 and E2F_TARGETS hallmarks followed this pattern of depletion in LumA/B cells, in line with previous work from Schulze et al*.* [50] and Oshi et al. [51, 52], respectively. Conversely, the ESTROGEN_RESPONSE_EARLY and ESTROGEN_RESPONSE_LATE hallmarks were enriched in LumA and B cells relative to other subtypes, as expected given the hormonal-driven nature of this subtype [53]. Her2 cells showed enrichment of the OXIDATIVE_PHOSPHORYLATION hallmark, a result supported by recent work examining expression of an oxidative phosphorylation gene signature across breast cancer subtypes [51, 52]. Similarly, for Basal cells, we found enrichment of the EPITHELIAL_MESENCHYMAL_TRANSITION and ALLOGRAFT_REJECTION hallmarks, both of which have been strongly linked to the Basal-like bulk tumor subtype [54] and tumors classified as TNBC by IHC [55], respectively. Heterogeneity was also observed between cell cycle phases within each subtype, with pathway changes aligning well with known biology. For example, the G2M_CHECKPOINT hallmark was depleted in G0/G1 and S and enriched in G2/M cells in all four subtypes and the MITOTIC_SPINDLE hallmark was depleted in G0/G1 and S and enriched in G2/M again, in all four subtypes except in LumA G0/G1 phase (Fig. 3c). Similarly, the MYC_TARGETS_V1 was depleted in G0/G1 and enriched in S and G2/M phases for Her2 and Basal cells, matching its known role in regulating S phase entry [56, 57]. These observations highlight the importance of considering both subtype-specific and cell cycle-dependent differences in cancer hallmark expression, laying the foundation for more refined analyses of potential therapeutic targets.

Beyond transcriptional differences, well-characterized genomic alterations also shape the signaling landscape of breast cancer subtypes. TP53 is most frequently mutated in Basal-like and HER2-enriched tumors, whereas PIK3CA mutations occur predominantly in Luminal subtypes, and BRCA1 germline variants are enriched in a subset of triple-negative tumors. Our results are broadly consistent with these established patterns and with previous large-scale analyses. In the TCGA breast cancer study [58], TP53 transcriptional activity signatures were highest in Luminal A tumors and lowest in Basal-like; similarly, Wu et al. reported that Luminal-skewed neoplastic gene modules were enriched for p53 signaling and apoptosis, whereas Basal-skewed modules were not [5]. These findings parallel our observation in GRN across subtype comparisons (Fig. 4b) that the “P53_PATHWAY” hallmark was most active in Luminal A/B cells, where TP53 is typically wild-type, and depleted in Basal-like cells, where TP53 mutations are common. Similarly, analyses by Madsen et al. [59] showed that PI3K-pathway signatures, including “MTORC1_SIGNALING”, were lowest in Luminal A and highest in Basal/HER2 tumors, and that PIK3CA-mutated ER⁺/HER2⁻ cancers displayed reduced mTORC1 activity. This somewhat counterintuitive pattern, where a pathway may be genetically altered yet show attenuated canonical target expression, mirrors our findings and highlights that mutation does not necessarily equate to increased downstream transcriptional activity. As the single-cell atlases analyzed here lack mutational profiles, we cannot directly link DNA-level changes to transcriptional activity; however, these parallels suggest that the observed pathway enrichments likely reflect both genomic context and subtype-specific cellular states. Integrating single-cell DNA and RNA sequencing in future studies will be critical to delineate how genetic lesions and transcriptional programs jointly contribute to pathway heterogeneity across breast cancer subtypes.

To place our results in a more treatment focused context we sought to identify compounds that could be candidates for targeting specific TF(regulon)-subtype-phase-pathway combinations. Here we highlight pertinent previously published data for a selection of drugs and chemicals but note that an exhaustive review of all compounds found in our analyses is beyond the scope of this work. Among these compounds, we found that Adapalene, a third-generation topical retinoid used to treat acne vulgaris, emerged as a candidate compound for hallmark pathways including HYPOXIA in Her2 cells in G0/G1 phase. Adapalene and other retinoids are notable as they have previously demonstrated promising anti-cancer potential and efficacy as therapeutic agents targeting breast cancer stem cells [60]. Similarly, Vinblastine, which disrupts the mitotic spindle apparatus, causing cellular arrest during mitosis and inducing apoptosis [61], and Irbesartan, an angiotensin receptor blocker used to treat hypertension that was shown to reduce metastasis in hepatocellular carcinoma [62], were also identified as candidate compounds targeting the HYPOXIA hallmark pathway in HER2 cells in the G0/G1 phase. Hallmarks including APOPTOSIS and P53_PATHWAY were found to be potentially targetable in LumA and B cells in S phase using the androgen and anabolic steroid Nandrolone decanoate. This steroid has, however, been tested in a clinical trial setting in the 1980s and did not improve overall response rate in advanced breast cancer when added to Tamoxifen [63, 64]. Whilst we only focused on FDA approved drugs in our results, we also found significant hits for chemicals in CTD. Acetylcysteine, for example, was found to target ESTROGEN_RESPONSE_EARLY and ESTROGEN_RESPONSE_LATE in LumA and LumB cells in G0/G1 phase, and is used as a mucolytic in patients with lung conditions and to treat acetaminophen overdose, and it has also demonstrated potential benefits in breast cancer [65–68]. These findings underscore the importance of considering not only subtype information but also cell cycle phases in drug targeting strategies. By integrating subtype-cell cycle-specific information, we can develop more effective, precision cancer medicine treatment approaches for breast cancer.

To showcase this potential application of our work, we propose a hypothetical situation where scRNA-seq analysis has been applied to a surgically removed breast tumor sample. We approach this scenario from an ideal future precision medicine perspective where treatments are only given on the basis of targets identified in tumor cells rather than the current standard of care [69, 70]. As in our analysis above, scRNA-seq is used to first provide a detailed map of the tumor’s heterogeneity, showing that it contains cells of multiple molecular subtypes in different cell cycle phases (see illustration in Fig. 6). Second, network analyses are used to derive specific subtype-phase enriched pathways where compound targets are identified. For this example, we focus on a tumor where the predominant single-cell subtypes are LumA and B (which is the most common case we saw in our two analyzed cohorts). A first option could utilize combination therapy, for a review, see [71]. Here, we could combine “Drug 1” and “Chemo 1” to target “Pathway 1” in LumA G0/G1 phase cells, “Chemo 2” to target “Pathway 2” in LumA G2/M phase cells, “Chemo 3” and “Drug 2” to target “Pathway 1” in LumB S phase cells, and “Drug 3” and “Chemo 4” to target “Pathway 3” in LumB G2/M cells (Fig. 6). A second strategy could be to employ sequential therapy, where a single chemotherapeutic agent or drug is administered per treatment course in a sequential fashion. This method may be particularly beneficial when taking cell cycle dynamics into account by arresting cells in specific phases before administering the subsequent chemotherapeutic agent to potentially enhance therapeutic efficacy. Going back to the same illustrative example, we could start with “Chemo 2” to target “Pathway 2” in LumA G2/M phase cells; this would arrest cells in a specific phase, introducing a non-arbitrary distribution of cells into the remaining phases. For instance, if the “Chemo 2” arrests cells in G2/M phase, that would mean that cells will be in either G0/G1 or S phase, reducing the variability of cell cycle phases and thus, optimizing the usage of the next compound. Subsequently, for the next sequential compound, in the illustrative example, only the “Chemo 3” and “Drug 2” would be needed for LumB cells in S phase. In general, combination therapy would be a more “bulk” treatment process where all relevant pathways could be targeted simultaneously, and sequential therapy would allow for a finer grain treatment where a more specific selection of drugs/chemo agents would be used. However, the toxicities associated with combination therapies would likely preclude their usefulness in our hypothetical approach.

There are also some limitations to this study. First, despite the plethora of tools available to infer the cell cycle phase of each cell [72], these methods typically lack generalizability and do not capture cell cycle dynamics properly [19]. Acknowledging these challenges, we relied on Seurat for cell cycle phase inference as it has been benchmarked and shown to be an effective and computationally efficient approach that is suitable for large cohorts. Second, we analyzed the G0 and G1 cell cycle phases together due to the challenge of accurately distinguishing between these transcriptionally similar states [72]. New studies have emerged that may be better able to separate these two phases [73], however, this methodology has not been benchmarked or tested against other computational methods. Third, this analysis represents a snapshot in time. Cells are assigned to a cell-cycle phase at measurement, but they will continue to progress through phases, so temporal stability within the same patient cannot be assessed without longitudinal data. In addition, clinical stage was not consistently available across datasets and was therefore not modeled; phase proportions and pathway activity may vary with stage and treatment context. Fourth, since our primary aim was to compare PAM50 subtypes and cell-cycle phases at the population level, we did not harmonize or explicitly model all clinical or genetic variables (for example BRCA1 status, treatment, or stage), and these factors may contribute to within-subtype heterogeneity and to variation in phase proportions and pathway activity with stage and treatment context. Fifth, this study is purely computational and relies on previously published single-cell RNA-seq datasets. We do not provide in vitro or in vivo validation of the inferred subtype- and phase-specific pathway signals or compound-TF(regulon)-pathway links. Future work should evaluate these predictions using subtype- and phase-resolved experimental models, for example, through perturbation of candidate transcription factors, pathway inhibition, or compound-response assays. Sixth, our analysis is confined to the transcriptional layer. The single-cell atlases used here do not include matched copy-number, epigenomic, or proteomic profiles per cell, and hallmark pathway activity can also be modulated by these additional regulatory layers. Our results should therefore be viewed as RNA-level evidence of subtype- and phase-specific pathway activity, which will need to be refined and tested in future single-cell multi-omic and proteomic studies. Despite this, we believe our results to be robust and representative of processes present during each phase—this is owing to the aforementioned methodology of combining cells across a broad number of tumors and using a second cohort to confirm all findings.

In conclusion, our study shows the importance of cell cycle phases in understanding breast cancer biology, increasing the biological insights compared to bulk analysis. The distinct landscape of biological mechanisms active in each subtype-phase specific scenario suggests that compound candidates targeting these pathways may offer a more targeted and effective approach to breast cancer treatment.

## Supplementary Information

Additional file 1 (PDF 39 KB)

Additional file 2 (PDF 487 KB)

Additional file 3 (PDF 18 KB)

Additional file 4 (PDF 62 KB)

Additional file 5 (XLSX 546 KB)

Additional file 6 (XLSX 34 KB)

Additional file 7 (DOCX 14 kb)

## Data Availability

The data used in this study are two publicly available breast cancer atlases, Wu et al. [5] and Pal et al. [30]. Code to reproduce the results of this study is publicly available at https://github.com/MiguelCastresana/bc_decoding_cellcycle

## Abbreviations

IHC: Immunohistochemistry
ER: Estrogen receptor
PR: Progesterone receptor
HER2: Human epidermal growth factor receptor 2
scRNA-seq: Single cell RNA-sequencing
DGE: Differential gene expression
FGSEA: Fast Gene Set Enrichment Analysis
TF: Transcription factor
GRN: Gene regulatory network
RSS: Regulon Specificity Score
CTD: Comparative Toxicogenomics Database
FDA: Food and Drug Administration
