# Supplementary material for: Computational decoding of cell-cycle phase effects on cancer hallmarks across breast cancer subtypes
Source: Breast Cancer Res. 2025 Dec 24;28:19. doi: 10.1186/s13058-025-02208-1 (PMC12849333; doi:10.1186/s13058-025-02208-1)
Supplement: Supplementary file 1 — Additional file 1 (PDF 39 KB) [file 13058_2025_2208_MOESM1_ESM.pdf]

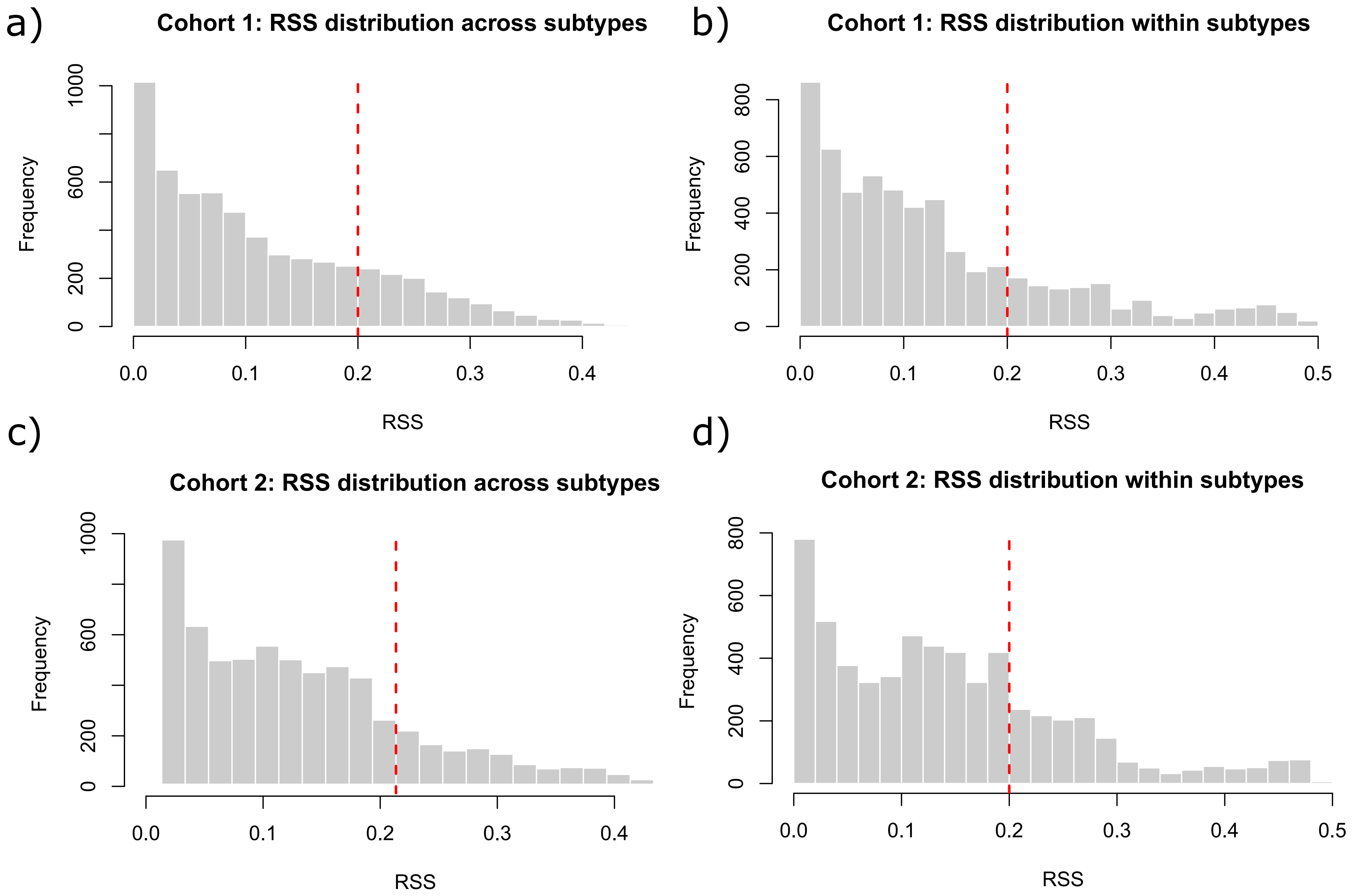
a)
Cohort 1: RSS distribution across subtypes
Frequency
0
200
600
1000
0.0
0.1
0.2
0.3
0.4
RSS
b)
Cohort 1: RSS distribution within subtypes
Frequency
0
200
400
600
800
0.0
0.1
0.2
0.3
0.4
0.5
RSS
c)
Cohort 2: RSS distribution across subtypes
Frequency
0
200
600
1000
0.0
0.1
0.2
0.3
0.4
RSS
d)
Cohort 2: RSS distribution within subtypes
Frequency
0
200
400
600
800
0.0
0.1
0.2
0.3
0.4
0.5
RSS
