# Supplementary material for: Computational decoding of cell-cycle phase effects on cancer hallmarks across breast cancer subtypes
Source: Breast Cancer Res. 2025 Dec 24;28:19. doi: 10.1186/s13058-025-02208-1 (PMC12849333; doi:10.1186/s13058-025-02208-1)
Supplement: Supplementary file 2 — Additional file 2 (PDF 487 KB) [file 13058_2025_2208_MOESM2_ESM.pdf]

a)

Sample	IHC	MFS	LumA %	LumB %	Her2 %	Basal %	G0/G1 %	S %	G2/M %
CID3941	ER+	LumA	53	39	3	5	59	26	16
CID4290A	ER+	LumA	92	5	1	1	55	39	7
CID4530N	ER+	LumA	99	0	0	0	46	35	19
CID3948	ER+	LumB	5	94	1	0	79	17	4
CID4067	ER+	LumB	23	74	2	1	56	36	8
CID4461	ER+	LumB	1	73	23	2	50	29	21
CID4463	ER+	LumB	30	57	12	0	55	30	15
CID4471	ER+	LumB	24	71	0	5	31	28	41
CID4535	ER+	LumB	0	99	0	0	60	29	11
CID3921	HER2+	Her2	0	0	100	0	74	18	8
CID4066	HER2+	Her2	28	15	56	1	58	28	13
CID45171	HER2+	Her2	0	0	97	2	58	24	18
CID3963	TNBC	Basal	11	30	7	52	71	14	16
CID4465	TNBC	Basal	1	0	26	73	10	22	69
CID4495	TNBC	Basal	0	0	0	100	41	38	21
CID44971	TNBC	Basal	0	0	1	99	55	26	19
CID4513	TNBC	Basal	5	8	16	72	51	24	26
CID4515	TNBC	Basal	0	0	0	100	51	27	22
CID44991	TNBC	Her2	2	2	92	4	53	29	18
CID4523	TNBC	Her2	11	2	68	19	38	38	23

b)

Sample	IHC	MFS	LumA %	LumB %	Her2 %	Basal %	G0/G1 %	S %	G2/M %
ER-0319	ER+	Basal	5	3	35	57	41	31	28
ER-0167-T	ER+	Her2	8	13	74	5	55	22	22
ER-0001	ER+	LumB	29	45	21	4	60	30	10
ER-0025	ER+	LumB	24	51	14	11	35	41	24
ER-0029-7C	ER+	LumB	9	84	5	2	57	31	12
ER-0029-9C	ER+	LumB	27	58	12	3	40	28	33
ER-0032	ER+	LumB	4	89	7	1	62	21	17
ER-0040	ER+	LumB	24	64	10	2	60	29	11
ER-0042	ER+	LumB	6	80	10	4	61	31	8
ER-0043-T	ER+	LumB	13	50	34	3	50	29	21
ER-0056-T	ER+	LumB	15	44	25	16	58	17	25
ER-0064-T	ER+	LumB	16	75	6	3	39	31	31
ER-0114-T3	ER+	LumB	18	69	11	3	39	42	19
ER-0125	ER+	LumB	15	71	9	5	50	33	17
ER-0151	ER+	LumB	10	76	11	2	61	20	19
ER-0163	ER+	LumB	31	47	18	5	68	20	12
ER-0173-T	ER+	LumB	18	55	24	3	60	26	14
ER-0360	ER+	LumB	14	53	29	4	61	26	12
HER2-0031	HER2+	Her2	2	12	86	0	51	32	17
HER2-0069	HER2+	Her2	0	1	96	3	53	30	18
HER2-0176	HER2+	Her2	6	14	76	4	68	16	17
HER2-0308	HER2+	Her2	4	41	53	2	60	28	11
HER2-0337	HER2+	Her2	3	4	89	4	53	26	21
HER2-0161	HER2+	LumB	4	56	38	1	61	20	19
TN-0106	TNBC	Basal	13	10	23	53	20	33	47
TN-0126	TNBC	Basal	0	0	7	93	43	39	18
TN-0135	TNBC	Basal	11	3	16	71	39	32	29
TN-B1-0131	TNBC	Basal	0	1	40	59	64	25	11
TN-B1-0177	TNBC	Basal	0	3	30	67	30	38	31
TN-B1-0554	TNBC	Basal	5	4	14	77	17	34	49
TN-B1-4031	TNBC	Basal	3	5	27	65	56	25	19
TN-0114-T2	TNBC	Her2	8	10	72	10	70	19	11
