# Supplementary material for: Computational decoding of cell-cycle phase effects on cancer hallmarks across breast cancer subtypes
Source: Breast Cancer Res. 2025 Dec 24;28:19. doi: 10.1186/s13058-025-02208-1 (PMC12849333; doi:10.1186/s13058-025-02208-1)
Supplement: Supplementary file 3 — Additional file 3 (PDF 18 KB) [file 13058_2025_2208_MOESM3_ESM.pdf]

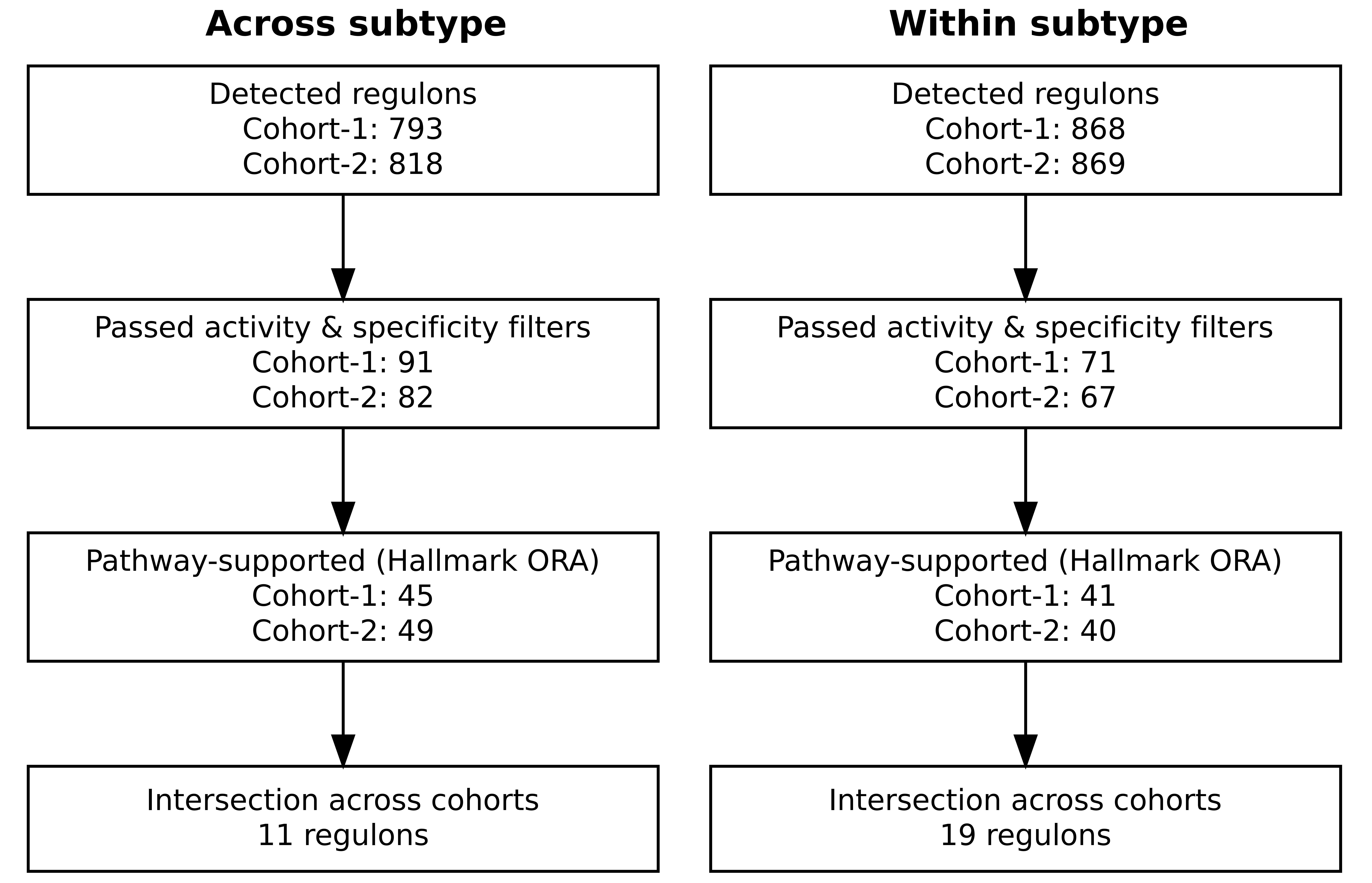
Across subtype
Within subtype
Detected regulons
Cohort-1: 793
Cohort-2: 818
Passed activity & specificity filters
Cohort-1: 91
Cohort-2: 82
Pathway-supported (Hallmark ORA)
Cohort-1: 45
Cohort-2: 49
Intersection across cohorts
11 regulons
Detected regulons
Cohort-1: 868
Cohort-2: 869
Passed activity & specificity filters
Cohort-1: 71
Cohort-2: 67
Pathway-supported (Hallmark ORA)
Cohort-1: 41
Cohort-2: 40
Intersection across cohorts
19 regulons
