# Supplementary figures and images for: Computational decoding of cell-cycle phase effects on cancer hallmarks across breast cancer subtypes
Source: Breast Cancer Res. 2025 Dec 24;28:19. doi: 10.1186/s13058-025-02208-1 (PMC12849333; doi:10.1186/s13058-025-02208-1)

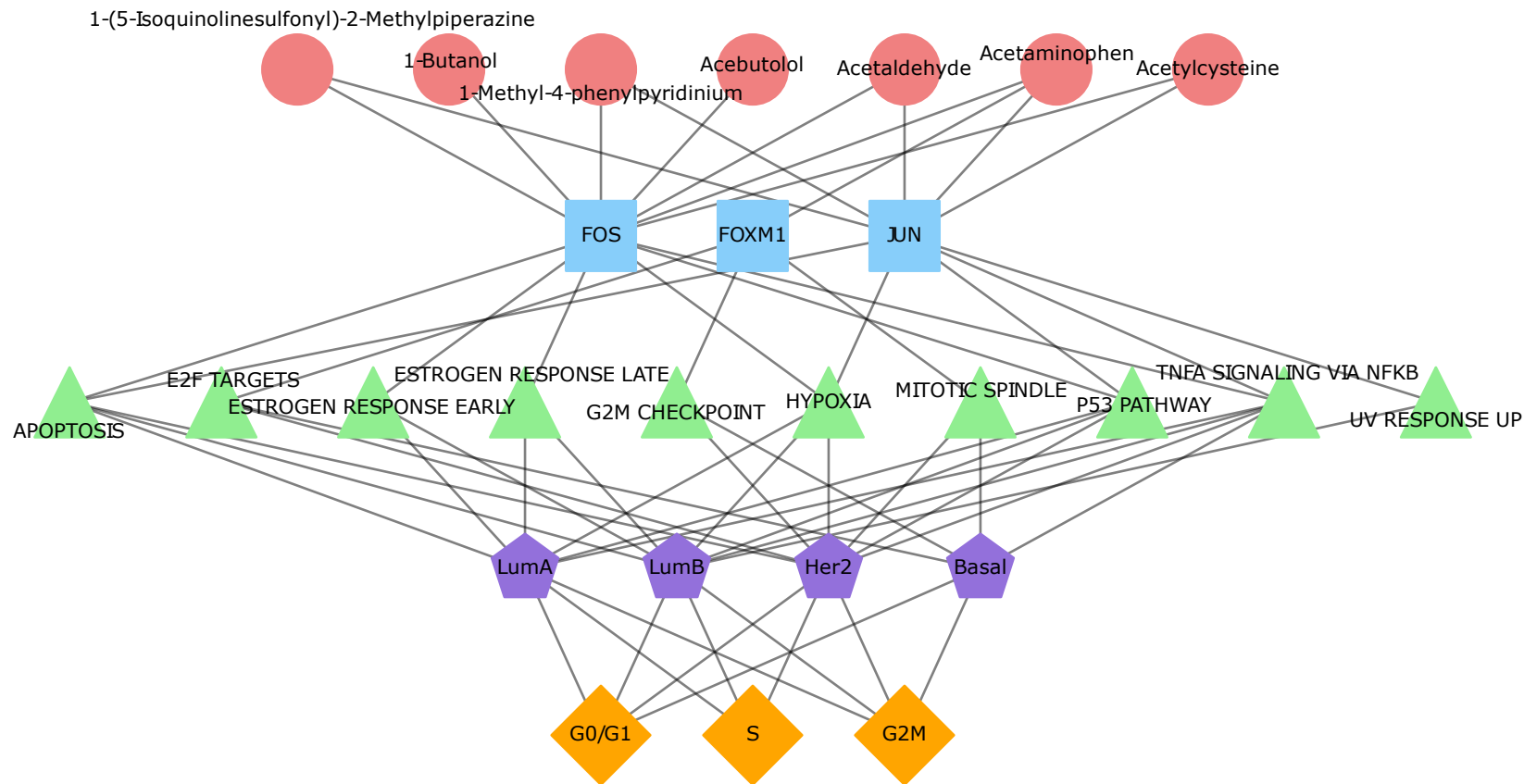

Supplement: Supplementary file 4 — Additional file 4 (PDF 62 KB) [file 13058_2025_2208_MOESM4_ESM.pdf]
