# Supplementary material for: Computational decoding of cell-cycle phase effects on cancer hallmarks across breast cancer subtypes
Source: Breast Cancer Res. 2025 Dec 24;28:19. doi: 10.1186/s13058-025-02208-1 (PMC12849333; doi:10.1186/s13058-025-02208-1)
Supplement: Supplementary file 7 — Additional file 7 (DOCX 14 kb) [file 13058_2025_2208_MOESM7_ESM.docx]

**Supplementary Table and Figure legends**

**Supplementary Table 1:** Results of FGSEA on ranked differentially expressed genes from the across subtype and within subtype analyses in cohort-1 and cohort-2. The table reports the FGSEA statistics (normalized enrichment score, p-value, and FDR-adjusted q-value).

**Supplementary Table 2:** Drug and chemical candidates targeting subtype- and phase-specific regulon–pathway combinations. FDA-approved drugs from DrugBank and chemicals from the Comparative Toxicogenomics Database (CTD) predicted to target transcription factors (regulons) are associated with Hallmark pathways with consensus enrichment in specific scPAM50 subtype and cell-cycle phase combinations. For each compound–TF–pathway–subtype–phase association, the table reports the compound name, source database, targeted transcription factor(s), associated pathway(s), and relevant identifiers. These entries correspond to the multipartite networks shown in Figure 5 and Supplementary Figure 4.

**Supplementary Figure 1:** Regulon Specificity Score (RSS) from the regulons obtained from the Gene Regulatory Networks (GRNs) inferred with SCENIC for both across subtypes and within subtypes analyses in cohort-1 and cohort-2.

**Supplementary Figure 2:** a) Discovery cohort with 20 samples and 24,489 cells. b) Validation cohort with 32 samples and 96,128 cells. MFS: Most Frequent.

**Supplementary Figure 3:** CONSORT-style diagram summarizing the multi-step filtering of regulons for the GRN analyses. For each cohort, we started from all regulons detected by SCENIC (“Detected regulons”), then retained those passing activity and subtype/phase-specificity filters (“Passed activity & specificity filters”) and finally selected regulons whose targets showed significant enrichment for at least one Hallmark pathway by ORA (“Pathway-supported”). The left branch shows counts for the across subtype analysis; the right branch shows counts for the within subtype analysis. The bottom boxes report the number of regulons that are reproducibly supported in both cohorts at the final stage of each analysis.

**Supplementary Figure 4:** A multipartite network with five distinct levels: Chemicals from Comparative Toxicogenomics Database (CTD), transcription factor target genes, target pathways, breast cancer subtypes, and cell cycle phases. Associations are represented as links.
